# Use of antibiotics in long-term care facilities for the elderly in Germany – point prevalence as a possible first step for data-based antibiotic stewardship

**DOI:** 10.3205/dgkh000472

**Published:** 2024-04-17

**Authors:** Ursel Heudorf, Kristin Stalla

**Affiliations:** 1 Institute for Hygiene and Environmental Medicine, Justus-Liebig University, Giessen, Germany; 2 Vivantes Forum for Seniors GmbH, Berlin, Germany

**Keywords:** long-term care facilities (LTCF) for the elderly, antibiotics, antibiotic consumption surveillance, antibiotic stewardship

## Abstract

**Introduction:**

In Germany, hospitals, rehabilitation facilities and outpatient surgery facilities are required by law to perform antibiotic-consumption surveillance. Established IT programs are available for recording the defined daily doses. These do not exist for long-term care facilities (LTCFs). Antibiotic stewardship is also recommended for LTCFs. In view of the lack of IT solutions for consumption surveillance, this study investigated whether point prevalence studies could be a suitable basis for a data-based antibiotic stewardship program in LTCFs.

**Method:**

In May 2023, 18 elderly-care facilities in Berlin, Germany, participated in a point prevalence survey on antibiotic consumption according to the established HALT (healthcare-associated infections in long-term care facilities) method. The number of residents present and their risk factors (including the use of catheters and antibiotic therapy) were recorded. The results were compared with comparable data from previous surveys in LTCFs in Berlin, Germany as well as with the HALT data for Europe as a whole and for Germany.

**Results:**

On the day of the survey, 2040 residents were present, 7.7% of whom bore a urinary catheter and 0.5% a vascular catheter. 0.2% of the residents had a port access, 0.4% a dialysis catheter and one resident (0.05%) a tracheostoma. Twenty-seven (1.3%) residents were receiving an antibiotic on the day of the survey. Of these, 29.6% had a urinary tract catheter. 63.0% of the antibiotics were given for a urinary tract infection, 14.8% for a respiratory tract infection and 11.1% for a wound/soft tissue infection. The overall prevalence of antibiotics was in the range of previous surveys from Germany (1.2–2.4%) and significantly lower than in the Europe-wide HALT survey overall (4.3–4.5%).

**Discussion:**

The survey showed low use of antibiotics in the LTCFs in comparison with Europe-wide surveys. The time required was less than 2 hours for a 100-bed facility. Until appropriate IT programs to determine the defined daily doses are also available for LTCFs, such easy-to-perform and standardized point-prevalence surveys – if repeated several times a year – can be a suitable method for recording the use of antibiotics in nursing homes for the elderly.

## Zusammenfassung

**Einleitung:**

In Deutschland sind Krankenhäuser, Rehabilitationseinrichtungen und Einrichtungen für ambulantes Operieren gesetzlich verpflichtet, eine Antibiotikaverbrauchssurveillance durchzuführen. Zur Erfassung der defined daily doses (DDD) liegen etablierte IT Programme vor. Diese gibt es nicht für Altenpflegeeinrichtungen. Auch für Altenpflegeeinrichtungen wird ein Antibiotic Stewardship empfohlen. Angesichts fehlender IT Lösungen für die Verbraus-Surveillance wird untersucht, ob Punktprävalenz-Studien eine geeignete Grundlage für ein datenbasiertes Antibiotic Stewardship Programm in Altenpflegeeinrichtungen sein können.

**Methode:**

Im Mai 2023 nahmen 18 Altenpflegeeinrichtungen in Berlin an den Punktprävalenzerhebungen zum Antibiotika-Verbrauch gemäß der etablierten HALT-Methode (healthcare associated infections in long term care facilities) teil. Dabei wurden neben den anwesenden Bewohnern auch deren Risikofaktoren, u.a. die Versorgung mit Kathetern und eine Antibiotikatherapie erfasst. Die Ergebnisse wurden mit vergleichbaren Daten aus früheren Erhebungen in Altenpflegeeinrichtungen in Deutschland sowie mit den HALT-Daten für Europa insgesamt und für Deutschland verglichen.

**Ergebnisse:**

Am Tag der Erhebung waren 2040 Bewohner anwesend. 7,7% waren mit einem Harnwegkatheter, 0,5% mit einem Gefäßkatheter versorgt. Einen Portzugang hatten 0,2% der Bewohner, einen Dialysekatheter 0,4% und ein Bewohner (0,05%) hatte ein Tracheostoma. 27 (1,3%) Bewohner erhielten am Tag der Erhebung ein Antibiotikum. Von diesen hatten 29,6% einen Harnwegkatheter. 63.0% der Antibiotikagaben erfolgten wegen einer Harnweginfektion, 14,8% wegen einer Atemweginfektion, 11,1% wegen einer Wund-/Weichteilinfektion. Die Antibiotika-Prävalenz lag insgesamt im Bereich früherer Erhebungen aus Deutschland (1,2–2,4%) und war deutlich geringer als in den europaweiten HALT-Erhebungen insgesamt (4,3–4,5%).

**Diskussion:**

Die Erhebung hat im Vergleich mit europaweiten Erhebungen einen niedrigen Antibiotikaeinsatz in den Altenpflegeeinrichtungen erbracht. Der Zeitaufwand betrug weniger als 2 h für ein 100-Betten-Haus. Bis entsprechende IT Programme zur Ermittlung der DDD auch für Altenpflegeeinrichtungen zur Verfügung stehen, können somit solche leicht durchführbaren und standardisierten Punkt-Prävalenz Erhebungen – bei mehrfacher Wiederholung im Jahr – als geeignete Methode zur Erfassung des Antibiotika-Einsatzes in Altenpflegeeinrichtungen Anwendung finden.

## Introduction

Given the increase in antibiotic-resistant pathogens, especially Gram-negative antibiotic-resistant pathogens, antibiotic stewardship programs are required in line with the one-health concept, not only in human but also in veterinary medicine [1], [2], [3], [4].

Against this backdrop, the German Antibiotic Resistance Strategy (DART) was implemented in Germany with the aim of reducing antibiotic consumption in animals and humans and thus also reducing the resistance pressure on bacterial pathogens [5], [6]. The effects of this strategy are published in reports on antibiotic consumption and resistance in animals and humans [7], [8]. The use of antibiotics in veterinary medicine is increasingly regulated by laws that restrict the quantities of antibiotics used and reserves certain antibiotics exclusively for human medicine. Antibiotic guidelines exist for the medical field (AB guidelines, e.g., urinary tract, etc.) [9], [10], [11], [12], and an S3 guideline on antibiotic stewardship has been published for the inpatient medical field, which contains structural specifications for the appropriate use of antibiotics [13]. According to Section 23 of the Infection Protection Act, hospitals, rehabilitation facilities, and outpatient surgery facilities in Germany are obliged to “continuously record data on the type and extent of antibiotic consumption in summarized form, evaluate it taking into account the local resistance situation, draw appropriate conclusions regarding the use of antibiotics, and ensure that the necessary adjustments to the use of antibiotics are communicated to the staff and implemented” [14].The exact method of recording was defined in publications from the Robert Koch- Institute [15], [16], [17].

Positive results are emerging. In veterinary medicine in Germany, the annual quantity of antibiotics dispensed fell from 1,731 tons in 2011 to 540 tons in 2022 [18]. In outpatient medicine, a decrease in prescriptions can be seen [19], [20], for instance, in the German federal state of Hesse, from 2,692,370 in 2011 to 1,708,134 prescriptions in 2020 [21]. Success is also noted in the clinical area [22], [23].

In recent years, the use of antibiotics in long-term care facilities for the elderly (LTCF) has also come under increasing attention. Following a pilot study in 2009, three prevalence studies on infections and antibiotic use in LTCFs, the so-called HALT studies (healthcare-associated infections in long-term care facilities in Europe), have now been conducted in Europe [24], [25], [26]. Data on the structural quality of the facilities, the risk profile of the care recipients (age, prevalence of medical devices or ulcers and wounds) and the prevalence of certain defined infections and antibiotic use are collected in the facilities on certain fixed dates. These show major quantitative and qualitative differences in the use of antibiotics in the various European countries [24], [25], [26].

Various studies, including major reviews and Cochrane publications, reveal that the use of antibiotics in LTCFs can be significantly reduced through training [27], [28], [29], but this was not associated with undertreatment, as hospital admissions and mortality did not increase [30]. These studies recommend antibiotic consumption surveillance (AVS) as a basis for evaluating the targeted improvements. However, there are no programs to date that can document antibiotic consumption in terms of defined daily doses (DDD)/resident day, as is the case in hospitals. In the following, the question of whether repeated point-prevalence studies may be suitable as an alternative for AVS will be investigated.

## Materials and methods

Eighteen (18) LTCFs in Berlin conducted point-prevalence surveys on antibiotic consumption in their facilities. The established HALT method was used [24], [25], [26]. Accordingly, on May 17^th^ 2023, the number of residents present in the respective facility was recorded, in addition to the proportion of residents aged 85 and over, men, residents with devices (transurethral and suprapubic urinary tract catheters, vascular catheters, port systems, dialysis catheters), and residents with systemic antibiotic therapy. For residents with antibiotic therapy, age, gender and devices were recorded individually, as well as the name of the antibiotic, the indication and any microbiological findings.

The results were analyzed descriptively (frequencies and odds ratios) and in a second step compared with comparable data from previous surveys in LTCFs in Germany as well as with the HALT data for Europe as a whole and for Germany.

## Results

On the day of the point-prevalence study, 2040 residents were present in the 18 facilities. Of these, 927 (45.4%) were over 85 years old, 765 (37.5%) were male, 157 (7.7%) were using a urinary catheter and 11 (0.5%) a vascular catheter. Five (0.2%) residents had a port access, 9 (0.4%) a dialysis catheter and one (0.05%) a tracheostoma. Twenty-seven (1.3%) residents were taking an antibiotic on the day of the survey (Table 1 (Tab. 1)). 

Among the residents with antibiotic therapy, 11.1% were over 85 years old, 51.9% were male and 29.6% had a urinary catheter. Compared to residents without antibiotic therapy, residents with antibiotic therapy were significantly younger (OR 0.147; 95% CI 0.044–0.492), more often male (OR 1.845; 95% CI 0.862–3.946, not significant) and were significantly more likely to have a urinary catheter (OR 5.267; 95% CI 2.268–12.235) (Table 1 (Tab. 1)).

In 17 (62.9%) residents, antibiotics were given for a urinary tract infection, 4 (14.8%) for a respiratory tract infection, 3 (11.1%) for a wound/soft tissue infection, one (3.7%) each for eye surgery and endocarditis in a heart valve. The indication was unknown for one resident (3.7%) receiving antibiotic treatment. Microbiological findings were documented for 4 residents, showing one case of *Staphylococcus aureus* pneumonia and three urinary tract infections, each with the occurence of *Pseudomonas aeruginosa *and *Proteus mirabilis*, and one with *Escherichia coli* in combination with a *Klebsiella* spp.

The number and distribution of antibiotics used are summarized by antibiotic class in Table 2 (Tab. 2). Penicillins with extended spectrum or in penicillin combinations were used most frequently (29.6%), followed by nitrofuran derivatives (25.9%) and third-generation cephalosporins, sulphonamides, tetracyclines and quinolones (7.4% each).

Table 3 (Tab. 3) shows the comparison of the data obtained in this survey in 2023 with results from earlier surveys using the identical recording method. Over the years, there has been a decrease in the use of urinary tract catheters in Germany, from over 10% to 7.7%. The antibiotic treatment prevalence of 1.3% was in the range of the results obtained from other LTCFs in Germany in the years 2009 to 2016 (1.2–2.4%), and was significantly lower than in European countries as a whole (4.3–5.4%). The current low use of quinolones is remarkable; in earlier studies up to 2016, it was still around 30% and therefore twice as high as the European average.

## Discussion

The presented point-prevalence study used the established method of the HALT surveys. The majority of the data could be retrieved from administrative data, while the questions on antibiotic therapy were obtained from the residents’ care files by the residential area managers or nursing staff. The time required for a 100-bed home was less than 2 hours. No special training of staff was needed, as the focus on antibiotic consumption meant that the more complex standardized survey of nosocomial infections with the query of defined infection symptoms was avoided. In principle, this procedure could therefore be carried out several times a year as antibiotic consumption surveillance and a data basis for antibiotic stewardship to be introduced in the future, also in nursing homes for the elderly. During these surveys, it should also be noted whether the duration of the prescribed antibiotic therapy has been set and recorded by the doctor, as a further quality feature of antibiotic use that is easy to record. On a positive note, the indications were noted for almost all antibiotic therapies. In contrast, a microbiological analysis was only available for 4 residents. However, it cannot be ruled out that the doctors had carried out more examinations, but the findings were documented in their files in the physician's practice and not in the care facility.

However, it might be desirable to record the DDD of the various active substances in the same way they are recorded in the clinics. Hospital pharmacies have simple programs to extract the quantities of active ingredients dispensed in micrograms from the defined pharmaceutical central numbers of the antibiotic packages dispensed and to specify them as Defined Daily Doses. This is then related to the patient days and stated as DDD/100 patient days. In principle, this procedure could also be applied to LTCFs in Germany. In Germany, residents of LTCFs are generally treated by GPs working in outpatient practices, who also prescribe the antibiotics. The LTCFs then order the antibiotics from their supply pharmacy, with some facilities being supplied by several pharmacies.

Pharmacies can easily compile the delivery of medicines from their administration file using the pharmaceutical central number (active ingredient, dose per tablet, dragée or milliliter and number of tablets per pack or total quantity in milliliters). So far, however, they lack a program that calculates DDDs from these data. A suitable program should be developed and made available to pharmacies free of charge. In contrast to hospitals, it cannot be completely ruled out in LTCFs that relatives may take the prescription to another pharmacy themselves. However, the LTCFs report that the proportion of relatives doing so is certainly very low and would not have a significant impact on the overall result.

Both methods, the point prevalence and the survey of total deliveries, have their advantages and disadvantages. While the survey of deliveries by pharmacies indicates the total quantity per year, a day with particularly high or low antibiotic use may have been selected at random in the case of point prevalence. However, this uncertainty can be reduced by repeating the survey several times per year. While no information on the indication for prescribing the antibiotic is recorded in the pharmacies’ delivery data, in the point-prevalence survey, the indication can be obtained from the residents' records. However, the further question of whether this indication and therapy are carried out in accordance with the guidelines cannot easily be answered by the nursing staff as part of the survey. This would require the support of the attending physicians, e.g., as part of an antibiotic stewardship program.

The prevalence of antibiotic prescriptions in our study did not show a decrease; it had been rather low in surveys in Germany for years. There is, however, an improvement in the use of antibiotics, as the proportion of quinolone prescriptions was significantly lower than in previous studies. This is consistent with the data from physicians’ practices and clinics [19], [20], [21], [22], [23]. There too – in the context of successful antibiotic stewardship – a slight reduction in overall prescriptions was achieved, in contrast to a significant reduction in prescriptions of quinolones, probably thanks to Direct Healthcare Professional Communications (“Dear Doctor” letters), which warned doctors of serious side effects of quinolones (aortic aneurysms, dissections or tendon ruptures) and demanded a restriction of their use for certain indications only [31], [32].

Compared with the data from the HALT studies from all European countries [24], [25], [26], the prevalence of antibiotic prescriptions is significantly lower in the Berlin facilities, but also in LTCFs in Germany that were examined earlier [33], [34], [35], [36], [37], [38]. Looking at the medical care of nursing-home residents across Europe, two-thirds of residents are cared for by general practitioners, in Germany almost exclusively by general practitioners. While approximately a quarter of facilities across Europe have doctors on their staff, this is the absolute exception in Germany. Across the EU, 60% [25] and 77.8% [26] of facilities had “coordinating physicians”, and a third of these physicians had the task of developping an antibiotic policy for the facility.

In contrast, only 38% [25] and 17% [26] of the facilities in Germany had coordinating physicians, albeit without the task of establishing an antibiotics policy. Nevertheless, in all HALT studies, not only the use of antibiotics but also the prevalence of infections in the participating homes in Germany was lower than the EU average. This may be due to the comparatively better hygiene structure in the facilities in Germany. For example, 88% of the facilities in Germany had hygiene specialists and 80% had a hygiene committee. Across Europe, only 71% of facilities had hygiene specialists and 39% had a hygiene committee [26]. The consumption of hand antiseptics in Germany, at 8.4 ml/resident day, was about twice as high as in facilities across Europe (4.3 ml/occupant day) [26]. Whether this can be attributed to the implementation of the recommendations on hygiene and infection prevention in care facilities for the elderly published by the Commission for Hospital Hygiene and Infection Prevention (KRINKO) [39] in 2005 remains to be answered.

## Conclusions

In view of the increase in antibiotic-resistant pathogens, a general reduction in antibiotic consumption and antibiotic stewardship is required in LTCFs. Various studies have shown that training for doctors and nursing staff in LTCFs can significantly reduce unnecessary antibiotic prescriptions [40]. The decrease in inappropriate antibiotic use was highest in studies that examined antibiotic use for urinary tract infection (UTI) in LTCFs [41]. In 2017, the Centers for Medicare & Medicaid Services (CMS) mandated long-term care facilities (LTCFs) to establish antimicrobial stewardship programs (ASPs) [42]. Various ways in which ABS can be implemented in LTCFs have been published [43], [44], [45]. The basis for assessing the measures is antibiotic consumption surveillance. The method of antibiotic point prevalence in elderly-care facilities presented in this publication can be carried out easily and without a great investment of time. Thus, if repeated several times a year, it can be used as a suitable method for recording the use of antibiotics in elderly-care facilities.

## Notes

### Competing interests

The authors declare that they have no competing interests.

### Author’s ORCID

- Ursel Heudorf: 0000-0002-0050-8272

## Figures and Tables

**Table 1:**
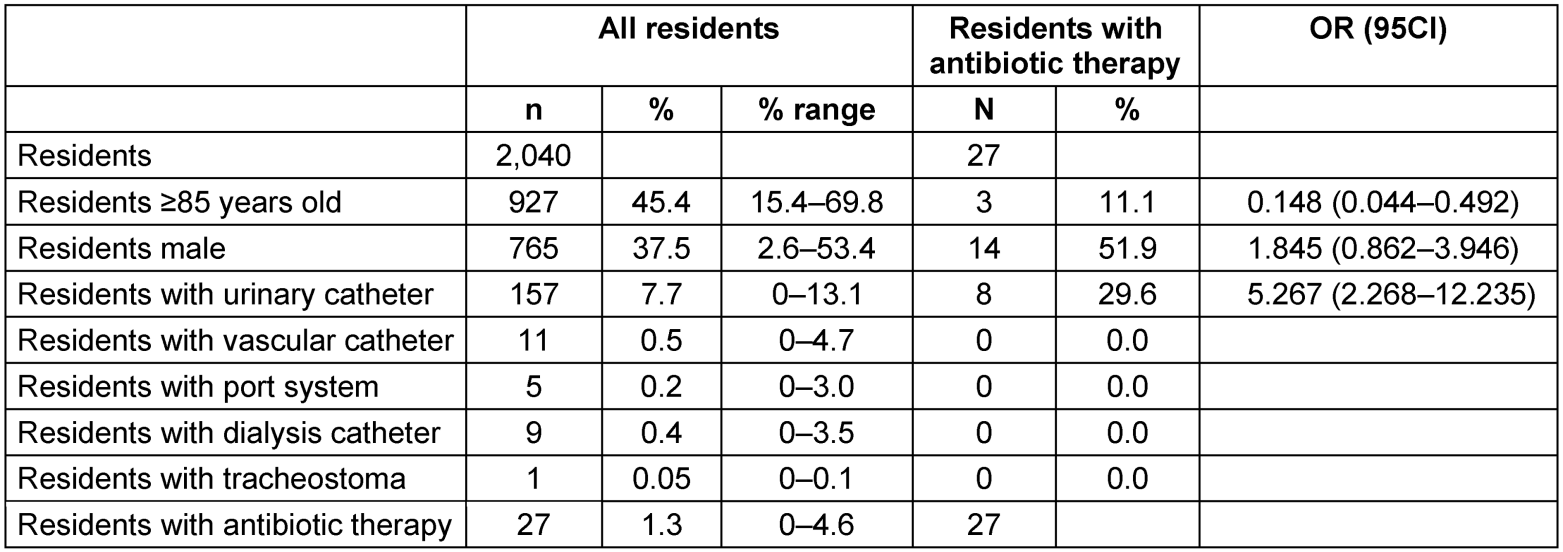
Age, gender, medical devices and antibiotic therapies of the 2,040 residents present on the survey day and included in the survey

**Table 2:**
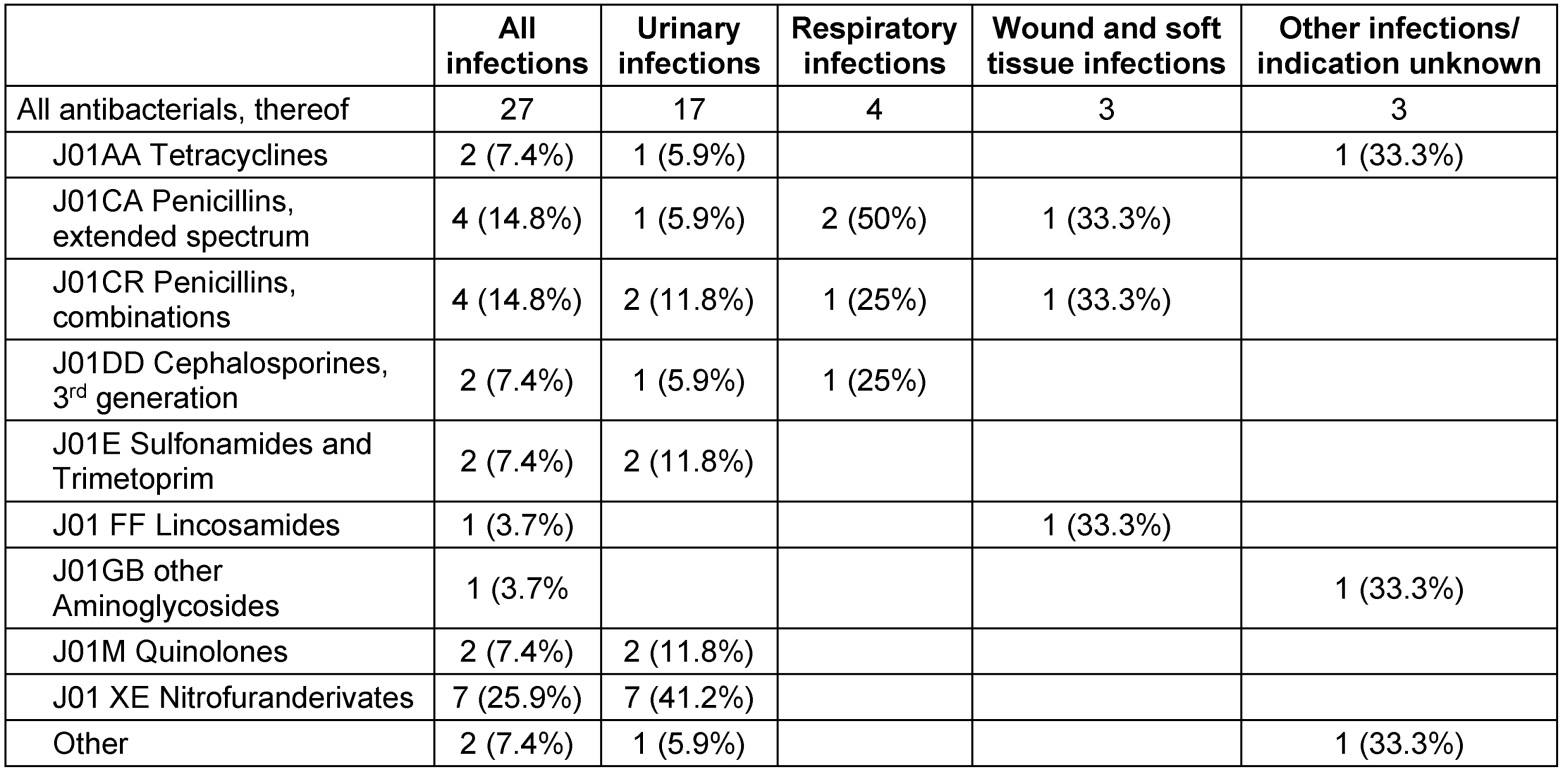
Active substance groups and indications for the antibiotics used in the 27 nursing home residents on the day of the survey

**Table 3:**
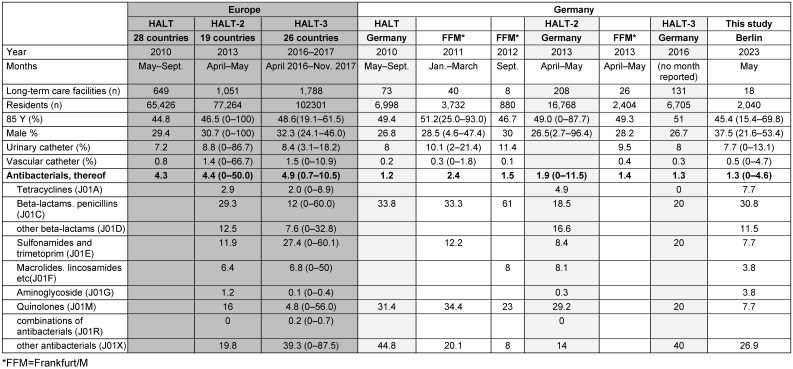
Personal characteristics, risk factors and antibacterial therapy in residents of long-term care facilities – data of the HALT Studies (health care associated infection in long-term care facilities) in Europe and of various studies in Germany compared with this study (Berlin 2023)
